# Longitudinal Study of Hepatitis A Infection by Saliva Sampling: The Kinetics of HAV Markers in Saliva Revealed the Application of Saliva Tests for Hepatitis A Study

**DOI:** 10.1371/journal.pone.0145454

**Published:** 2015-12-21

**Authors:** Luciane Almeida Amado Leon, Adilson José de Almeida, Vanessa Salete de Paula, Renata Santos Tourinho, Daniel Antunes Maciel Villela, Ana Maria Coimbra Gaspar, Lia Laura Lewis-Ximenez, Marcelo Alves Pinto

**Affiliations:** 1 Laboratory of Technological Development in Virology, Oswaldo Cruz Institute Foundation, Rio de Janeiro, RJ, Brazil; 2 Laboratory of Viral Hepatitis, Oswaldo Cruz Institute Foundation, Rio de Janeiro, RJ, Brazil; 3 Scientific Computing Program Department-PROCC, Oswaldo Cruz Institute Foundation, Rio de Janeiro, RJ, Brazil; Kaohsiung Medical University Hospital, Kaohsiung Medical University, TAIWAN

## Abstract

Despite the increasing numbers of studies investigating hepatitis A diagnostic through saliva, the frequency and the pattern of hepatitis A virus (HAV) markers in this fluid still remains unknown. To address this issue, we carried on a longitudinal study to examine the kinetics of HAV markers in saliva, in comparison with serum samples. The present study followed-up ten patients with acute hepatitis A infection during 180 days post diagnosis (dpd). Total anti-HAV was detected in paired serum and saliva samples until the end of the follow-up, showing a peak titer at 90^th^. However, total anti-HAV level was higher in serum than in saliva samples. This HAV marker showed a probability of 100% to be detected in both serum and saliva during 180 dpd. The IgM anti-HAV could be detected in saliva up to 150 dpd, showing the highest frequency at 30^th^, when it was detected in all individuals. During the first month of HAV infection, this acute HAV marker showed a detection probability of 100% in paired samples. The detection of IgM anti-HAV in saliva was not dependent on its level in serum, HAV-RNA detection and/or viral load, since no association was found between IgM anti-HAV positivity in saliva and any of these parameter (p>0.05). Most of the patients (80%) were found to contain HAV-RNA in saliva, mainly at early acute phase (30^th^ day). However, it was possible to demonstrate the HAV RNA presence in paired samples for more than 90 days, even after seroconversion. No significant relationship was observed between salivary HAV-RNA positivity and serum viral load, demonstrating that serum viral load is not predictive of HAV-RNA detection in saliva. Similar viral load was seen in paired samples (on average 10^4^ copies/mL). These data demonstrate that the best diagnostic coverage can be achieved by salivary anti-HAV antibodies and HAV-RNA tests during 30–90 dpd. The long detection and high probability of specific-HAV antibodies positivity in saliva samples make the assessment of salivary antibodies a useful tool for diagnosis and epidemiological studies. The high frequency of HAV-RNA in saliva and the probability of detection of about 50%, during the first 30 dpd, demonstrate that saliva is also useful for molecular investigation of hepatitis A cases, mainly during the early course of infection. Therefore, the collection of saliva may provide a simple, cheap and non-invasive means of diagnosis, epidemiological surveys and monitoring of hepatitis A infection purposes.

## Introduction

Hepatitis A virus (HAV) is the most common agent causing acute liver disease with approximately 1.4 million of new cases occurring every year worldwide [1]. It is a significant health problem in primary school due to the clinical aspect of the disease where asymptomatic patients contribute to disease spread among children, causing outbreaks that can be propagated to community. Studies have shown that in developing countries improvements in sanitary conditions have led to a reduction in childhood exposure to HAV, resulting in an increased disease burden in older population groups [2,3] and also several outbreaks [4,5].

Very sensitive and specific serological tests for detection of HAV antibodies are widely available for serum samples. They are key tools for diagnosis, epidemiological knowledge and prevention strategy whereby infected or susceptible persons can be identified, and directed to a medical care or vaccination program in order to prevent or reduce further transmission in these populations. However, many studies have shown that saliva have a great potential as alternative tool for diagnosis of several viral diseases, such as by human immunodeficiency virus (HIV) [6,7], hepatitis A [8,9,10], hepatitis B [11], hepatitis C [12,13], and herpes simplex [14].

The collection of saliva as an alternative to blood collection offers potential advantages for diagnosis and epidemiological studies. Due to its minimally invasive and simple collection, saliva testing can reduce discomfort, a major concern to children, thereby simplifying diagnosis and the collection of serial samples for monitoring disease states over time. Besides, it allows a noninvasive investigation of HAV subclinical cases, which occur frequently among children, and facilitates HAV vaccination candidates tracing. Thus, in recent years, has aroused a lot of interest in saliva as source of HAV antibodies and viral detection for diagnosis purposes and monitoring hepatitis A.

However, the concentration of IgG in saliva has been reported to be substantially lower (average 300 times) when compared to its concentration in serum [15,16,17]. Many studies have reported sensitivity and specificity rates comparable to those observed in blood-based tests [8,9,18,19], while others have demonstrated problems with assay sensitivity due to the low antibody levels in saliva [20]. Then the lower antibodies titers in saliva may become undetectable earlier than in serum.

Despite few reports evidenced the presence of HAV RNA in oral fluid [9,21], the frequency and load of HAV in it remains still controversial [22,23,24]. The knowledge of the true frequency of oral carriage of HAV and if the levels of the virus in oral fluids correlate with those of blood, may be central to suggest oral fluids as a vehicle of the transmission of hepatitis A and also to determine if saliva can be a tool to conduct molecular epidemiological studies of HAV.

These inconsistent data warrant the need for well-planned longitudinal studies to explore the precise frequency and pattern of anti-HAV antibodies and HAV-RNA in oral fluid in comparison with serum, in order to stablish if saliva may be useful for epidemiological studies, diagnosis and monitoring of hepatitis A. In particular, this approach is very important for intermediate endemic regions where HAV vaccination is critical to control the large outbreak of epidemics. Thus, the aim of this study was to describe the serological and virological course of hepatitis A infection through a longitudinal analysis of salivary antibodies and viral load pattern.

## Material and Methods

### Studied population

Patients attending to the Viral Hepatitis Ambulatory (VHA) of Institute Oswaldo Cruz, Rio de Janeiro, Brazil, during May 2009 to January 2010, with signs and/or symptoms of acute viral hepatitis such as jaundice, fever, general malaise, fatigue, nausea, vomiting, and anorexia, were screened for the study. After laboratory diagnosis of hepatitis A by detection of serum IgM anti-HAV antibodies, 10 patients were invited to participate into the longitudinal follow-up, where consecutive blood and saliva specimens were collected at 0, 30, 90, 120, 150, and 180 days post diagnosis (dpd). No sample was collected before the onset of illness. The ‘0th’day was defined as the day of hepatitis A diagnosis.

### Ethical aspects

Ethical permission for collecting and testing samples was provided by the FIOCRUZ Ethical Committee (536/09). Before entering into the study, all subjects or their guardians, on behalf of the minors/children enrolled in the study, have signed a written informed consent, previously approved by the Fiocruz ethical committee. The specimens were anonymous feedback was given to all participants of the study, including their results.

### Biological samples

All saliva samples were collected using OraSure collection devices (Epitope Incorporated, Beaverton, OR, USA) according to the manufacturer’s instructions. Samples were later recovered by centrifugation at 2500×*g* for 15 min at 10°C and stored at −20°C until analysis. Total blood samples were collected by venipuncture into sterile tubes, after which serum was obtained by centrifugation and stored at −20°C. Serum samples were conventionally used for serological and molecular diagnosis of HAV infection, and considered as the ‘gold standard’ for comparison with saliva tests [25].

### Liver function

Laboratory determination of serum alanine aminotransferase (ALT) and aspartate aminotransferase (AST) levels were carried out by a manual colorimetric procedure using a commercially available kit (Abbott, North Chicago, IL, USA).

### Detection of specific anti-HAV antibodies in serum and saliva

Serum IgM and total anti-HAV antibodies were detected by using enzyme immunoassays (Biokit, Barcelona, Spain) according to the manufacturer’s instructions. For saliva samples, undiluted samples (100 μL and 15 μL for IgM and total anti-HAV, respectively) were tested in a modified ELISA (Biokit, Barcelona, Spain) [9]. The cut-off point for HAV antibody detection was evaluated based on receiver operating characteristic curve (ROC curve) analysis (MedCalc for Windows, version 7.6.0.0, MedCalc Software, Mariakerke, Belgium). The ability of the model to differentiate between positive and negative individuals for total and IgM anti-HAV antibodies (discrimination) was quantified using the area under the curve (AUROC) test [26,27].

### Detection and quantification of HAV-RNA in serum and saliva samples

HAV-RNA was extracted using the QIAamp^™^ viral RNA extraction kit (Qiagen, Germany). A TaqMan real-time PCR assay was performed to quantify HAV genomes as previously described by de Paula et al. [28]. Viral RNA was amplified using primers derived from the most conserved region of HAV genome, the 5’ non-coding region (5’ NCR). The cDNA synthesis was carried out using 10 μL of RNA (10 pg– 5 μg), 10 pmol/μL of anti-sense primer (HAV5NCRI-R), 1 U/μL of RNasin (Promega, Madison, WI), 125 mM of each deoxynucleoside triphosphate, and 1 U/μL of Superscript III (Invitrogen, Carlsbad, CA, USA), in a final volume of 20 μL at 50°C for 1 h, followed by incubation for 10 min at 65°C. After cDNA synthesis, a master mix containing 1x TaqMan Universal PCR Master Mix (Applied Biosystems, Hammonton, NJ, USA) and 1.25 μL of the assay mixture (300 nM each primer, 150 nM probe) (Applied Biosystems Assay, Foster City, CA, USA) was prepared. Five microliters of cDNA and standard curve points were added to 20 μL of the PCR master mix. The thermal cycling conditions were as follows: an initial step at 50°C for 2 min and 95°C for 10 min, followed by 40 cycles at 95°C for 15 s and at 60°C for 1 min. The primers and probe used to quantify the 5′NTR are described by de Paula et al. [28].

### Statistical Analysis

We used Chi-square test and student’s *t*-test as appropriate. The Mann–Whitney *U*-test was used to evaluate differences between continuous variables (age and viral load). The diagnostic value of anti-HAV antibodies assay in saliva samples was assessed by receiver operating characteristic (ROC) curve. An area under curve (AUC) of 1.0 is characteristics of an ideal test, whereas 0.5 indicates a test of no diagnostic value. All data were analyzed by two tailed tests and a *p* value less than 0.05 was considered significant.

The kinetics of total and IgM anti-HAV titers, and viral load were analyzed as separate univariate models by applying Generalized Additive Model with Mixed effects (GAMM), where the time after illness onset was considered as explanatory variable using a smoothing spline and random effects per patient, with Gaussian error distribution and identity link function. After fitting models we found mean values and 95% confidence intervals to describe kinetics for both saliva and serum samples. In order to estimate probabilities of test positivity at days after illness onset for each of the diagnostic outcomes (total anti-HAV titre, IgM anti-HAV titre, and viral load), we take the binary outcomes (positive/negative) from each of the test results and their counts were used as observations for fitting logistic regression models. Number of days after illness onset and sample type (saliva/serum) are explanatory variables and a log link function is used. Analyzes were performed using R software [29] and package GAMM4 [30].

## Results

### Demographic characteristics of studied population

Ten patients with acute hepatitis A, aged 3–28 years (14.3 **±** 7.6 years), five of them were males, was included in this study. All participants were followed up to 180 days, except for patient 10 (p10) who was followed up until 120 days.

### Anti-HAV antibodies response and biochemical profile during the follow-up of HAV cases

**Fig 1:**
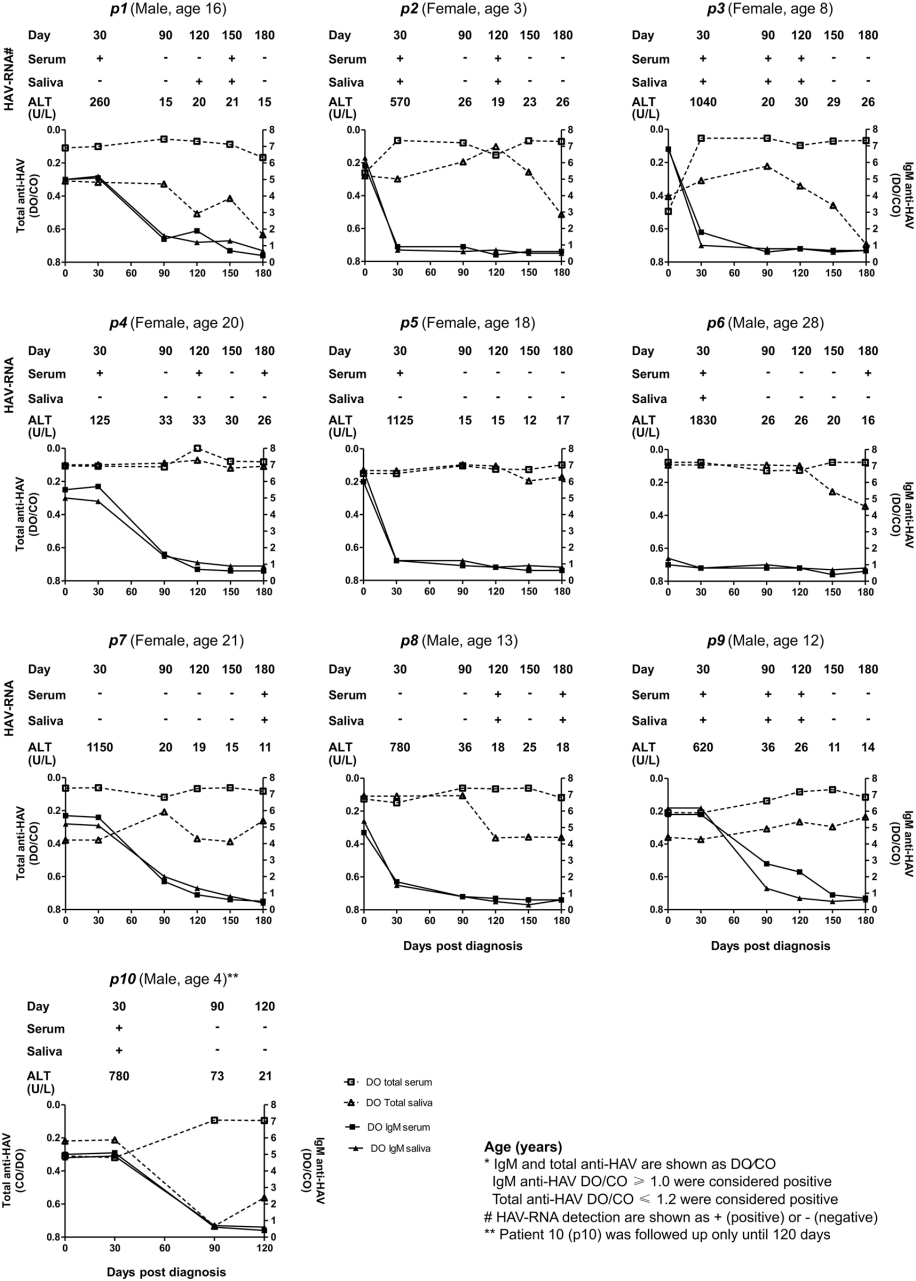
Individual analysis of the profile of anti-HAV antibodies response and HAV-RNA detection in saliva samples comparing to serum during the follow-up. Age (years); *IgM and total anti-HAV are shown as DO/CO; IgM anti-HAV DO/CO ≥ 1.0 were considered positive; Total anti-HAV DO/CO ≤ 1.2 were considered positive; ^#^ HAV-RNA detection are shown as + (positive) or—(negative); ** Patient 10 (p10) was followed up only until 120 days.

In Fig 1, the individual analysis of HAV antibodies profile in paired serum and saliva samples, and serum ALT levels are presented for all patients (p1 –p10), during the follow-up period. In all individuals, ALT levels were elevated during the first 30 days, but declined to baselines values afterwards. The outcome for total anti-HAV tests was positive for all individuals in both saliva and serum up to the end of the study. However, in most of the patients (7/10) total anti-HAV OD/CO was higher in serum than in saliva samples during the course of infection.

The IgM anti-HAV was detected in saliva of all individuals at 30^th^ and patients were likely to remain IgM positive in paired serum and saliva samples, up to day 120. However, in five individuals (p1, p4, p5, p6 and p7), salivary anti-HAV IgM remained detectable for longer than in serum samples (Fig 1) (S1 File).

**Fig 2:**
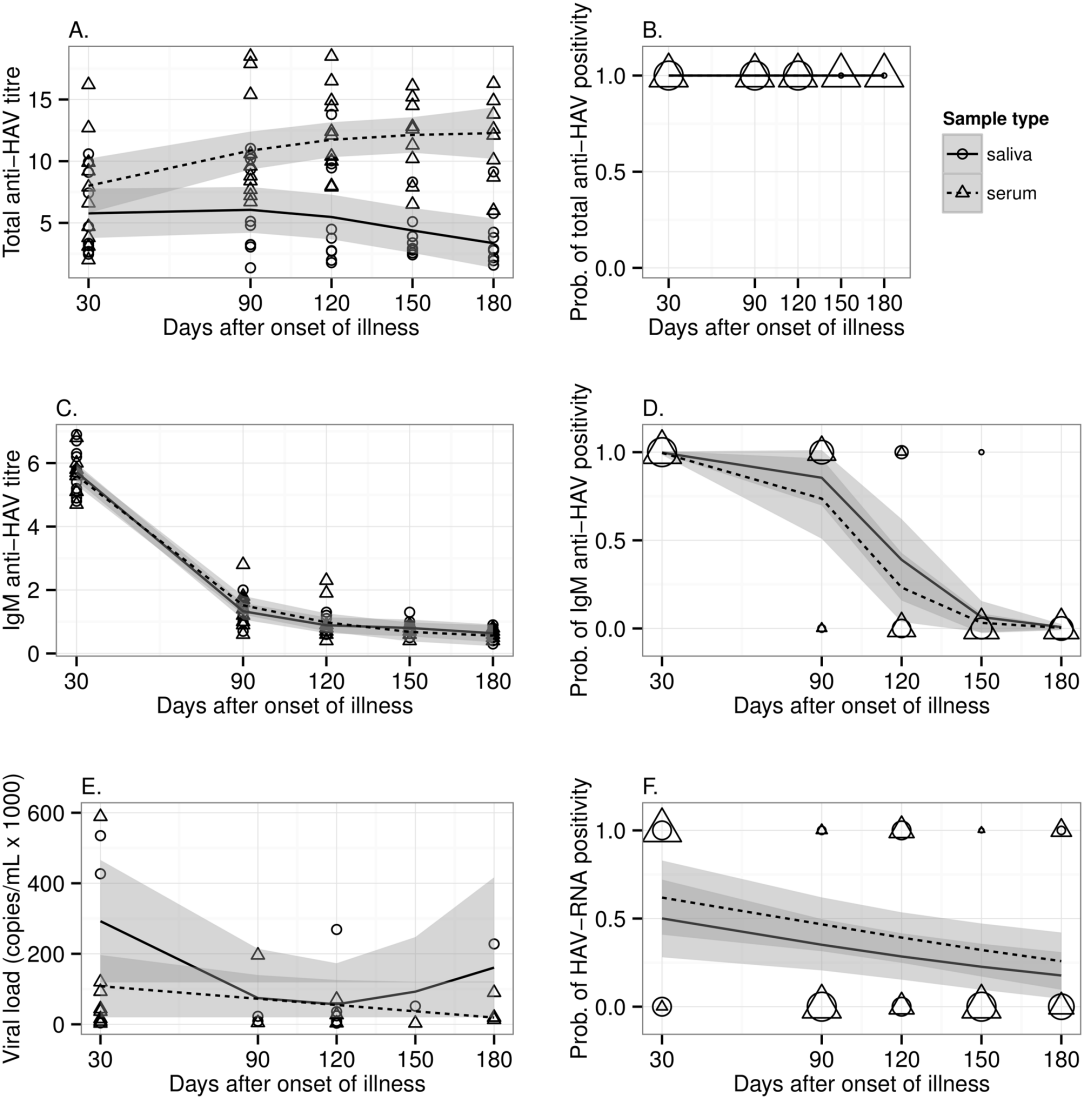
Course of hepatitis A in patients followed during 180 days by serum and salivary diagnostic assays: observations for total anti-HAV (A), IgM anti-HAV (C), and viral load (E) and probabilities of positivity for total anti-HAV (B), IgM anti-HAV (D) and HAV-RNA (F). Solid lines and shaded areas along them represent for saliva the predicted mean values and 95% confidence intervals, whereas dashed lines and respective shaded areas represent for serum results the predicted mean values and 95% confidence intervals. In A-C-E, circles and triangles represent individual observations for saliva and serum, respectively, and in B-D-F, represent the counts of positive tests (value 1) or negative (value 0) for saliva and serum, respectively. Shape sizes (circles and triangles) are proportional to the counts.

Fig 2 shows the overall kinetics of serum and saliva IgM and total specific-antibodies and the probability of these markers detection along the course of infection. IgM and total anti-HAV was detected already on the 30^th^, the earliest serum and saliva samples. The kinetics of total anti-HAV in saliva showed a peak of the titer on the 90^th^, followed by a decreasing trend of the titers afterwards. While in serum samples, total anti-HAV titer tend to increase until the end of the follow-up, and in general, this titer was higher in serum than in saliva samples (Fig 2A). This HAV marker showed a probability of 100% to be detected in both serum and saliva during 180 dpd (Fig 2B).

Along the course of infection, IgM anti-HAV demonstrated a similar pattern of response in paired specimens. During the first month of HAV infection, this acute HAV marker showed a detection probability of 100% in paired samples. On the 90^th^, 80% of the saliva and serum samples were still positive for IgM anti-HAV, with a probability of 85% (95% CI: 70–100%) and 74% (95% CI: 50–96%) for IgM anti-HAV detection in saliva and serum, respectively. Overall, patients were likely to remain IgM positive in paired serum and saliva samples, up to illness day 120, with a probability of 24% (95% CI: 4–43%) and 39% (95% CI: 16–62%), respectively (Fig 2D).

No significant difference was observed between serum and saliva IgM anti-HAV OD/CO (median = 1.88 in serum, and 1.87 in saliva) (*p* = 0.28) (Fig 2C). To determine a relative correlation between the level of IgM anti-HAV in saliva and its level in serum during the follow up, a statistical approach was used. From days 30–90 post onset, there was a high positive correlation between the optical density (OD) values in serum and saliva (r = 0.997 [95% CI = 0.97–0.99] p < 0.001). While, from days 120–180, a lower correlation was seen (r = 0.74 [95% CI = 0.29–0.92] p < 0.0056).

The association of IgM anti-HAV detection in saliva samples was evaluated with IgM antibody level [DO/CO] in serum, HAV-RNA detection and viral load in serum and saliva. Each parameter was grouped in two categories: IgM level (DO/CO): ≤ 5.0 and >5.0; HAV-RNA detection: positive and negative; viral load: ≥ 20.000 and < 20.000. No association was found between IgM anti-HAV positivity in saliva and any parameter (Fisher test, p>0.05 for each comparison).

### Comparison of HAV-RNA detection and viral load between serum and saliva samples

In order to determine the duration of HAV detection and viral load in saliva, serial paired serum and saliva samples from 10 HAV patients were evaluated. HAV-RNA was more frequently detected during the early course of infection (at 30^th^) in serum (8/10) and in saliva (6/10) samples (Fig 1). An intermittent pattern of HAV RNA detection was seen in some individuals both in serum (p1, p2, p4, p6 and p8) and in saliva (p2 and p8) samples. Most of the patients positive for HAV RNA in saliva (8/10) also showed viremia. However, two patients (p4 and p5) were negative for HAV-RNA in saliva during all the follow up, and showed viremia. A delayed HAV-RNA detection was seen in patients p7 and p8 (from 180 and 120 dpd, respectively), in both kind of specimens, persisting detectable up to the end of follow-up (Fig 1). The mean viral load ranged from 10^3^ to 10^5^ copies/mL, in paired samples (Table 1) (S1 File), reaching a peak at the 30^th^, followed by a decreasing trend up to the end of follow up (Fig 2E).

**Table 1:** Comparison of viral load between serum and saliva samples during the follow-up of hepatitis A cases.

patient	Time points (dpd)									
	30		90		120		150		180	
	serum	saliva	serum	saliva	serum	saliva	serum	saliva	serum	saliva
p1	8.4x10^3^	ND	ND	ND	ND	3.5x10^4^	3.0x10^3^	5.1x10^4^	ND	ND
p2	1.5x10^4^	5.3x10^5^	ND	ND	2.6x10^4^	8.8x10^3^	ND	ND	ND	ND
p3	4.3x10^4^	9.8x10^3^	4.4x10^3^	8.9x10^3^	3.6x10^3^	3.3x10^3^	ND	ND	ND	ND
p4	3.6x10^4^	ND	ND	ND	2.6x10^4^	ND	ND	ND	1.9x10^4^	ND
p5	9.3x10^4^	ND	ND	ND	ND	ND	ND	ND	ND	ND
p6	2.8x10^3^	3.9x10^3^	ND	ND	ND	ND	ND	ND	1.3x10^4^	ND
p7	ND	ND	ND	ND	ND	ND	ND	ND	1.9x10^4^	3.5x10^3^
p8	ND	ND	ND	ND	1.2x10^4^	2.4x10^4^	ND	ND	9.0x10^4^	2.2x10^3^
p9	5.8x10^5^	4.2x10^5^	1.9x10^5^	2.2x10^4^	7.1x10^4^	2.7x10^5^	ND	ND	ND	ND
p10	1.2x10^5^	2.3x10^5^	ND	ND	ND	ND	ND	ND	ND	ND
Mean (SD)	1.1x10^5^ (1.9x10^5^)	2.0x10^5^ (2.5x10^5^)	7.1x10^4^ (9.2x10^4^)	1.5x10^4^ (1.0x10^4^)	3.1x10^4^ (2.8x10^4^)	6.8x10^4^ (1.2x10^5^)	3.0x10^3^	5.1x10^4^	4.0x10^4^ (4.2x10^4^)	2.2x10^4^
Total positive cases	8/10	6/10	3/10	2/10	4/10	5/10	1/10	1/10	3/10	1/10

ND: Non Detected

Viral load: copies/mL

SD: Standard Deviation

During the follow up, HAV RNA was detected in saliva with a probability ranging from 50% (95% CI: 28–72%) (at 30^th^) to 18% (95% CI: 4–31%), at the end of the study. While the probability to detect viremia ranged from 62% (at 30^th^) (95% CI: 41–83% to 26% at the end of the study (95% CI: 10–42%) (Fig 2F).

To clarify whether the detection of HAV-RNA in saliva is dependent on the patients’ characteristics, i.e. antibodies levels, ALT and AST levels, HAV-RNA detection and/or viral load, the association of HAV-RNA positivity in saliva samples was evaluated with patients’ characteristics (gender, age, ALT level, IgM antibody level [CO/DO], serum HAV-RNA detection, and serum and saliva viral load). Each parameter were grouped in two categories: gender: F and M; age (years): ≤ 5 and > 5; ALT level (U/L): 40–1000 and >1000; IgM level (CO/DO): ≤ 5.0 and > 5.0; serum detection: + and -; serum viral load (copies/mL): ≤ 20.000 and > 20.000; saliva viral load (copies/mL): ≤ 20.000 and > 20.000. No association was found between HAV-RNA positivity in saliva and any patient characteristic (Fisher test, p>0.05 for each comparison).

## Discussion

Owing to the lower concentration of saliva IgM and IgG, and the controversial data about HAV-RNA detection in saliva, it is important to know the frequency and length of these HAV markers in the saliva samples to determine the feasibility and applicability of this fluid for diagnosis and epidemiological studies. Our previous studies demonstrated the detection of HAV specific-antibodies and HAV-RNA in saliva samples [9,31] of acute HAV cases. This further study is to the best of our knowledge, the first study to monitor the patterns of saliva immune response and genome detection during the course of hepatitis A infection, aiming to stablish if the saliva may be an alternative tool for the diagnosis, monitoring and epidemiological study of hepatitis A.

Although oral fluid collection has many advantages, when compared with total blood collection by venipuncture, some problems may arise if the choice of assay is not appropriate to detect antibodies in saliva samples. In the present study, an optimized assay was used to obtain optimal conditions for the detection of IgM and IgG anti-HAV antibodies in oral fluid [9]. In addition, a reduction of cut off value by approximately 18% was established, based on ROC curve analysis, to improve the test accuracy. The importance of establishing a cut-off ratio for the detection of HAV antibodies in oral fluid specimens has been demonstrated to be essential to increase the test accuracy [10].

During the course of HAV infection, it was demonstrated that total anti-HAV could be detectable in all patients, for at least 180 days after illness onset. However, a decreasing trend of the salivary total anti-HAV titer after 180^th^, suggests that, unlike the serum, it can become undetectable in saliva samples subsequently. The total anti-HAV DO/CO values were significantly lower in saliva, which confirms the lower titers of HAV antibodies in saliva [17]. This HAV marker showed a detection probability of 100%, until the end of the study. The long detection and high probability of total anti-HAV positivity in saliva samples make the assessment of salivary total anti-HAV a useful tool for diagnosis and epidemiological studies.

The profile of IgM anti-HAV response were quite similar between paired serum and saliva samples. However, in five individuals, salivary anti-HAV IgM remained detectable for longer that in serum samples. The longer detection of salivary anti-HAV IgM in these patients than in serum could be a reflects of a higher viraemia in serum samples, which may results in large amounts of IgM anti-HAV complexed with viral particles, becoming undetectable by serological assays. The difference of anti-HAV antibodies detection observed between patients was probably due the different phase of infection. Since the samples were collected from the day of hepatitis A diagnosis (‘0th’ day), the following days of collection did not represent a precise day of the infection. IgM anti-HAV could be detected in both types of samples from all subjects, on average, until 90 (ranging from 0 to 150) dpd. This finding is in accordance with a follow-up study conducted in 29 acute-phase patients that indicated that IgM anti-HAV persisted for 2–4 months and were not usually detectable thereafter [32].

Considering the good correlation (r = 0.99, p< 0.001) between the optical density (OD) values of the paired samples, the high frequency (100%-80%) and probability (100%-85%) of IgM anti-HAV detection in saliva of HAV acute cases at 30 and 90^th^ (100%-80%, respectively); the salivary IgM anti-HAV test showed a potential of substitute serum test in diagnosis of hepatitis A infection, mainly during the first 90 days post infection. No association was seen between HAV antibodies detection in saliva and antibodies level in serum, or the HAV RNA detection and/or viral load, indicating that the detection of anti-HAV antibodies in saliva is not dependent on these parameters.

Most of the hepatitis A patients (80%) were found to contain HAV RNA in saliva, as reported earlier [9,21,33]. HAV-RNA was often detected at early acute phase (during the first 30 days) of the infection in serum and saliva samples. However, it was possible to demonstrate the viral persistence in paired samples for more than 90 days, even after seroconversion. These current findings revealed a longer saliva and serum viremia than that reported by Costa-Mattioli et al. [34] who demonstrated persistent viremia, for an average of 60 days after clinical onset. The different period of HAV-RNA detection among patients must reflect the different stages of infection, since in some patients HAV-RNA was eliminated early (after 30 days) and in others it could be detected lately (as of 150 days). However, some authors suggested that the duration of viremia is dependent on the immunological and/or biochemical profile of the host [35]. In the present study, considering that, during some time points of the infection, HAV viral load was higher in saliva from some patients than in serum, and no significant relationship was observed between salivary HAV-RNA positivity and serum viral load (p = 0.4 95% IC = 0.33–1.0), these findings show that viral load in serum is not a predictive factor of HAV-RNA detection in saliva, as has been reported early by Amado et al. [9]. A previous study suggested that the HAV viral load in saliva might reflect a local site of HAV replication, as has been demonstrated a HAV replication in salivary gland of experimentally infected cynomolgus monkeys [31]. Therefore, an active HAV replication in the salivary glands would perhaps explain the discrepant HAV detection between matched serum and saliva specimens observed on day 120, when there were more positive cases in saliva than in serum.

Based on our results, similar viral load levels were seen in paired serum and saliva samples from each patient (on average 10^4^ copies/mL). Previous human HAV infections investigations have showed a mean serum viral load of 10^3^ copies/ml [34,36]. The 2-log difference between serum and saliva samples reported by Mackiewicz et al. [21] was not observed in the present study, which could be probably attributed to the use of different methods of quantification and different timing of sample collection [37]. The kinetics of HAV-RNA detection in both serum and saliva showed an intermittent pattern of detection in some patients, probably due the fluctuation in the viral load, which has also been demonstrated by Amado et al. [31] in cynomolgus monkeys experimentally infected with HAV.

Taken together, the high frequency of HAV-RNA in saliva and the probability of detection of about 50%, during the first 30 dpd, demonstrate that saliva is useful for molecular investigation of hepatitis A cases, mainly during the early course of infection. Nonetheless, the intermittent pattern of detection and low probability of HAV-RNA detection, in paired samples, after the first month of infection, suggests that HAV-RNA detection is not a sufficient diagnostic tool for the whole time period. The relationship between HAV detection and infectivity has not been established yet and, in order to evaluate the potential risk of transmitting HAV by saliva among patients during early acute phase or convalescence period, it would be interesting to conduct experimental infection studies in animal models [34,38].

In summary, these data demonstrate that saliva samples could be used as a method of detecting anti-HAV antibodies and HAV genome, and that the best diagnostic coverage can be achieved by salivary IgM and total antibodies detection during the first 90 dpd, which quite correlates with serum methods. Therefore, the collection of saliva may provide a simple, inexpensive and non-invasive means of diagnosis, epidemiological studies and monitoring of hepatitis A infection purposes.

## Supporting Information

S1 File Individual data of anti-HAV antibodies, ALT and HAV-RNA in serum and saliva, according the day of diagnosis (dpd). Age (years); IgM and total anti-HAV are shown as DO/CO; IgM anti-HAV DO/CO ≥ 1.0 were considered positive; Total anti-HAV DO/CO ≤ 1.2 were considered positive; ALT: alanine aminotransferase; Viral load (copies/mL); ND: Not detected.
(DOC)
