# Supplementary material for: Longitudinal Study of Hepatitis A Infection by Saliva Sampling: The Kinetics of HAV Markers in Saliva Revealed the Application of Saliva Tests for Hepatitis A Study
Source: PLoS One. 2015 Dec 21;10(12):e0145454. doi: 10.1371/journal.pone.0145454 (PMC4686585; doi:10.1371/journal.pone.0145454)
Supplement: S1 File — Age (years); IgM and total anti-HAV are shown as DO/CO; IgM anti-HAV DO/CO ≥ 1.0 were considered positive; Total anti-HAV DO/CO ≤ 1.2 were considered positive; ALT: alanine aminotransferase; Viral load (copies/mL); ND: Not detected. (DOC) [file pone.0145454.s001.doc]

| **Patient** | **Year** | **dpd** | **ALT/GLT (U/L)** | **DO/CO IgM serum** | **DO/CO IgM saliva** | **DO/CO total serum** | **DO/CO total saliva** | **viral load serum** | **viral load saliva** |
| --- | --- | --- | --- | --- | --- | --- | --- | --- | --- |
| p 1 | 16 | 30 | 260 | 5,1 | 5,2 | 9,9 | 3,2 | 8400 | ND |
|  |  | 90 | 15 | 1,4 | 1,6 | 17,9 | 3,1 | ND | ND |
|  |  | 120 | 20 | 1,9 | 1,2 | 14,4 | 2,0 | ND | 35800 |
|  |  | 150 | 21 | 0,7 | 1,3 | 11,3 | 2,4 | 3020 | 51700 |
|  |  | 180 | 15 | 0,4 | 0,7 | 6,0 | 1,6 | ND | ND |
| p 2 | 3 | 30 | 570 | 5,8 | 6,3 | 3,8 | 3,3 | 15700 | 535000 |
|  |  | 90 | 26 | 0,9 | 0,7 | 15,4 | 5,1 | ND | ND |
|  |  | 120 | 19 | 0,9 | 0,6 | 12,4 | 9,8 | 26800 | 8800 |
|  |  | 150 | 23 | 0,4 | 0,7 | 6,5 | 3,9 | ND | ND |
|  |  | 180 | 26 | 0,6 | 0,5 | 14,9 | 1,9 | ND | ND |
| p3 | 8 | 30 | 1040 | 0,6 | 0,5 | 14,1 | 2,1 | 43800 | 9805 |
|  |  | 90 | 20 | 6,8 | 6,9 | 2,0 | 2,5 | 4480 | 8914 |
|  |  | 120 | 30 | 1,8 | 1,0 | 18,5 | 3,2 | 3650 | 3356 |
|  |  | 150 | 29 | 0,6 | 0,8 | 18,5 | 4,5 | ND | ND |
|  |  | 180 | 26 | 0,8 | 0,8 | 10,2 | 2,9 | ND | ND |
| p4 | 20 | 30 | 125 | 0,7 | 0,6 | 13,8 | 2,2 | 36600 | ND |
|  |  | 90 | 33 | 0,7 | 0,7 | 14,7 | 1,4 | ND | ND |
|  |  | 120 | 33 | 5,7 | 4,8 | 9,2 | 9,9 | 26800 | ND |
|  |  | 150 | 30 | 1,6 | 1,5 | 8,8 | 11,0 | ND | ND |
|  |  | 180 | 26 | 0,7 | 1,1 | 10,0 | 13,8 | 19900 | ND |
| p5 | 18 | 30 | 1125 | 0,6 | 0,9 | 12,6 | 8,3 | 93000 | ND |
|  |  | 90 | 15 |  | 0,9 | 12,1 | 9,1 | ND | ND |
|  |  | 120 | 15 | 6,0 | 6,7 | 6,6 | 7,4 | ND | ND |
|  |  | 150 | 12 | 1,2 | 1,2 | 9,6 | 10,2 | ND | ND |
|  |  | 180 | 17 | 0,9 | 1,2 | 8,0 | 9,5 | ND | ND |
| p6 | 28 | 30 | 1830 | 0,8 | 0,8 | 7,9 | 5,1 | 2880 | 3940 |
|  |  | 90 | 26 | 0,6 | 0,9 | 10,1 | 5,8 | ND | ND |
|  |  | 120 | 26 | 0,6 | 0,8 | 12,9 | 4,6 | ND | ND |
|  |  | 150 | 20 | 5,6 | 5,8 | 12,7 | 10,6 | ND | ND |
|  |  | 180 | 46 | 1,0 | 1,4 | 7,7 | 10,5 | 13200 | ND |
| p7 | 21 | 30 | 1150 | 0,8 | 0,8 | 7,9 | 10,0 | ND | ND |
|  |  | 90 | 20 | 0,8 | 1,0 | 12,8 | 3,9 | ND | ND |
|  |  | 120 | 19 | 0,8 | 0,8 | 12,6 | 2,9 | ND | ND |
|  |  | 150 | 15 | 0,4 | 0,7 | 14,9 | 3,2 | ND | ND |
|  |  | 180 | 11 | 5,6 | 5,1 | 16,2 | 2,6 | 19100 | 3508 |
| p8 | 13 | 30 | 780 | 1,7 | 2,0 | 8,4 | 4,8 | ND | ND |
|  |  | 90 | 36 | 0,9 | 1,3 | 14,9 | 2,7 | ND | ND |
|  |  | 120 | 18 | 0,6 | 0,8 | 16,1 | 2,6 | 12156 | 24100 |
|  |  | 150 | 25 | 0,5 | 0,4 | 12,1 | 3,8 | ND | ND |
|  |  | 180 | 18 | 4,7 | 5,4 | 7,9 | 9,1 | 90000 | 228000 |
| p9 | 12 | 30 | 620 | 1,7 | 1,5 | 6,7 | 9,3 | 589000 | 427000 |
|  |  | 90 | 36 | 0,8 | 0,8 | 16,5 | 2,8 | 196000 | 22800 |
|  |  | 120 | 26 | 0,7 | 0,5 | 15,2 | 2,8 | 70900 | 269000 |
|  |  | 150 | 11 | 0,6 | 0,3 | 16,3 | 2,8 | ND | ND |
|  |  | 180 | 14 | 0,6 | 0,6 | 8,4 |  | ND | ND |
| p10 | 4 | 30 | 780 | 5,8 | 6,2 | 4,7 | 2,7 | 120000 | 238700 |
|  |  | 90 | 73 | 2,8 | 1,3 | 7,2 | 3,2 | ND | ND |
|  |  | 120 | 21 | 2,3 | 0,7 | 12,1 | 3,8 | ND | ND |
|  |  | 150 |  | 0,9 | 0,5 | 14,5 | 3,4 | ND | ND |
|  |  | 180 |  | 0,7 | 0,6 | 8,7 | 4,2 | ND | ND |

**S1 File. Individual data of anti-HAV antibodies, ALT and HAV-RNA in serum and saliva, according the day of diagnosis (dpd)**

Age (years)

IgM and total anti-HAV are shown as DO⁄CO

IgM anti-HAV DO/CO ≥ 1.0 were considered positive

Total anti-HAV DO/CO ≤ 1.2 were considered positive

ALT: alanine aminotransferase

Viral load (copies/mL)

ND: Not detected
